# Short- and long-term outcomes in onco-hematological patients admitted to the intensive care unit with classic factors of poor prognosis

**DOI:** 10.18632/oncotarget.7986

**Published:** 2016-03-08

**Authors:** Etienne Faucher, Martin Cour, Vincent Jahandiez, Adeline Grateau, Thomas Baudry, Romain Hernu, Marie Simon, Jean-michel Robert, Mauricette Michallet, Laurent Argaud

**Affiliations:** ^1^ Hospices civils de Lyon, Groupement hospitalier Edouard Herriot, Service de réanimation médicale, F-69003, Lyon, France; ^2^ Université de Lyon, Université Lyon 1, Faculté de médecine Lyon Est, F-69008, Lyon, France; ^3^ Hospices civils de Lyon, Centre hospitalier Lyon-Sud, Service d'hématologie, F-69310, Pierre-Bénite, France

**Keywords:** hematological malignancy, allogeneic hematopoietic stem cell transplantation, neutropenia, invasive mechanical ventilation, intensive care unit

## Abstract

Although the overall mortality of patients admitted to intensive care units (ICU) with hematological malignancy has decreased over the years, some groups of patients still have low survival rates. We performed a monocentric retrospective study including all patients with hematological malignancy in a ten-year period, to identify factors related to the outcome for the whole cohort and for patients with allogeneic hematopoietic stem cell transplantation (HSCT), neutropenia, or those requiring invasive mechanical ventilation (IMV). A total of 418 patients with acute leukemia (n=239; 57%), myeloma (n=69; 17%), and lymphoma (n=53; 13%) were studied. Day-28 and 1-year mortality were 49% and 72%, respectively. The type of disease was not associated with outcome. The disease status was independentlty associated with 1-year mortality only. Independent predictors of day-28 mortality were IMV, renal replacement therapy (RRT), and performance status. For allogeneic HSCT recipients (n=116), neutropenic patients (n=124) and patients requiring IMV (n=196), day-28 and 1-year mortality were 52%, 54%, 74% and 81%, 78%, 87%, respectively. Multivariate analysis showed that IMV and RRT for allogeneic HSCT recipients, performance status and IMV for neutropenic patients, and RRT for patients requiring IMV were independently associated with short-term mortality (p<0.05).

These results suggest that IMV is the strongest predictor of mortality in hematological patients admitted to ICUs, whereas allogeneic HSCT and neutropenia do not worsen their short-term outcome.

## INTRODUCTION

The incidence of hematological malignancies has recently been evaluated in Europe as 230,000 new cases per year, with an increasing use of intensive care unit (ICU) resources [1, 2]. As a result, intensivists are increasingly faced with managing these patients. The prognosis of onco-hematological patients admitted to ICUs has constantly improved over the last two decades [3]. Progress in diagnostic strategies of acute respiratory failure, in using non-invasive mechanical ventilation (NIMV), and advances in the treatment of the underlying malignancy help to explain this survival gain [4–6]. Consequently, admission policies have become less restrictive and ICUs are able to accept these patients [7].

However, some groups of patients still have a low survival rate. Numerous studies have identified predictors of hospital mortality including neutropenia, hematopoietic stem cell transplantation (HSCT), severity of illness, and organ supports [8–12]. Nevertheless, several concerns can be raised. First, prognostic factors evolve over time, which may lead to conflicting results for studies carried out at different periods. Second, in these previous studies, patients with hematological malignancy were not systematically separated from all cancer patients. However, it is well established that their prognostic factors and outcomes are different [13]. Third, as recently confirmed by Azoulay et al., autologous HSCT needs to be dissociated from allogeneic HSCT [12]. Finally, data concerning the long-term outcome of these patients are scarce [3].

Therefore, we conducted this single center retrospective study of a large cohort to assess the recent outcome of patients with hematological malignancy. We focused on both the short- and long-term outcomes of three subgroups of patients with both clinical relevance and classic low survival rate. Thus, we assessed the prognostic factors of patients with neutropenia, allogeneic HSCT, or those requiring invasive mechanical ventilation (IMV). A better understanding of these particular subgroups of patients may help in their management by ICU clinicians.

## RESULTS

### Characteristics and outcome of the study population

A total of 418 patients met the inclusion criteria. Patient characteristics, reasons for ICU admission, organ failures, and day-28 outcome are shown in Table 1. More than 60 patients were admitted in each 2-year period of the study timeframe (Table 2). Age, sex, breakdown of malignancies, SAPS II, use of IMV, and mortality rates were not significantly different across the five periods (Table 2).

**Table 1:** Patient characteristics according to day-28 outcome

	All patients (n=418)	Survivors (n=215)	Non-survivors (n=203)	Univariate analysis p Value	Multivariate analysis p Value
Age^a^	55 ± 15	53 ± 16	57 ± 15	0.01	0.22
Sex (male)^b^	244 (58)	128 (60)	116 (57)	0.62	
Charlson score^a^	4.1 ± 2.1	3.9 ± 2.1	4.4 ± 2.1	0.03	0.37
PS^a^	1.8 ± 1.0	1.7 ± 1.0	2.1 ±1.0	<0.001	<0.01
Hematological malignancies^b^					
Type				0.35	
Acute leukemia	239 (57)	124 (58)	115 (57)		
Myeloma	69 (17)	34 (16)	35 (17)		
Lymphoma	53 (13)	32 (15)	21 (10)		
Chronic leukemia	27 (6)	14 (7)	13 (6)		
Others	30 (7)	11 (5)	19 (9)		
Disease status				0.06	0.84
Newly diagnosed	129 (31)	63 (29)	66 (33)		
Controlled/Remission	136 (33)	81 (38)	55 (27)		
Recurrence/Progression	156 (37)	71 (33)	82 (40)		
Treatment/Condition					
Autologous HSCT	43 (10)	26 (12)	17 (8)	0.21	
Allogeneic HSCT	116 (28)	56 (26)	60 (30)	0.42	
Neutropenia	124 (30)	57 (27)	67 (33)	0.15	
Reasons for admission^b^				0.02	NA
Respiratory	199 (48)	92 (43)	107 (53)		
Hemodynamic	112 (27)	69 (32)	43 (21)		
Metabolic	63 (15)	36 (17)	27 (13)		
Neurologic	29 (7)	11 (5)	18 (9)		
Cardiac arrest	6 (1)	1 (0)	5 (2)		
Others	9 (2)	6 (3)	3 (1)		
Organ failures^b^				<0.0001	NA
n = 0-1	205 (49)	147 (68)	58 (29)		
n = 2-3	192 (46)	66 (31)	124 (61)		
n ≥ 4	21 (5)	2 (1)	19 (9)		
SOFA score^a^	8 ± 4	7 ± 3	10 ± 3	<0.0001	NA
Organ supports^b^^c^					
IMV	196 (47)	51 (24)	145 (71)	<0.0001	<0.0001
RRT	99 (24)	26 (12)	73 (36)	<0.0001	<0.001
Vasopressors	214 (51)	89 (41)	125 (62)	<0.0001	0.82
SAPS II^a^	58 ± 24	46 ± 16	70 ± 26	<0.001	NA
Length of stay in ICU^a^	10 ± 15	12 ± 19	7 ± 6	<0.001	NA

a Data expressed as mean ± SD.

b Data expressed as number (percentage).

c Within the first 48 hours HSCT: hematopoietic stem cell transplantation; ICU: intensive care unit; IMV: invasive mechanical ventilation; NA: not applicable; NS: not significant; PS: performance status; RRT: renal replacement therapy; SAPS II: simplified acute physiology score II; SOFA: sequential organ failure assessment.

**Table 2:** Evolution of patients' characteristics and outcome during the study period

	2002-2003 (n=77)	2004-2005 (n=108)	2006-2007 (n=61)	2008-2009 (n=83)	2010-2011 (n=89)	p Value
Age^a^	53 ± 14	54 ± 15	57 ± 14	55 ± 17	57 ± 17	0.16
Sex (male)^b^	40 (52)	65 (60)	38 (62)	47 (57)	54 (61)	0.52
Hematological malignancies^b^						
Acute leukemia	35 (45)	72 (67)	33 (58)	51 (61)	48 (54)	0.11
Myeloma	17 (22)	18 (17)	10 (16)	15 (18)	9 (10)	0.33
Lymphoma	12 (16)	12 (11)	6 (10)	7 (8)	16 (18)	0.31
Treatment/Condition^b^						
Allogeneic HSCT	20 (26)	32 (30)	19 (31)	20 (24)	25 (28)	0.81
Neutropenia	20 (26)	33 (31)	16 (26)	29 (35)	26 (29)	0.79
IMV^c^	38 (49)	56 (52)	25 (41)	36 (43)	41 (46)	0.87
SAPS II^a^	52 ± 22	58 ± 25	57 ± 14	61 ± 26	59 ± 20	0.24
Day-28 mortality^b^	37 (48)	59 (55)	26 (43)	42 (51)	39 (44)	0.75
Day-90 mortality^b^	43 (56)	67 (62)	32 (52)	49 (59)	51 (57)	0.43
1-year mortality^b^	53 (69)	82 (76)	41 (67)	60 (72)	66 (74)	0.96

a Data expressed as mean ± SD.

b Data expressed as number (percentage).

c Within the first 48 hours HSCT: hematopoietic stem cell transplantation; IMV: invasive mechanical ventilation; SAPS II: simplified acute physiology score II.

**Figure 1:**
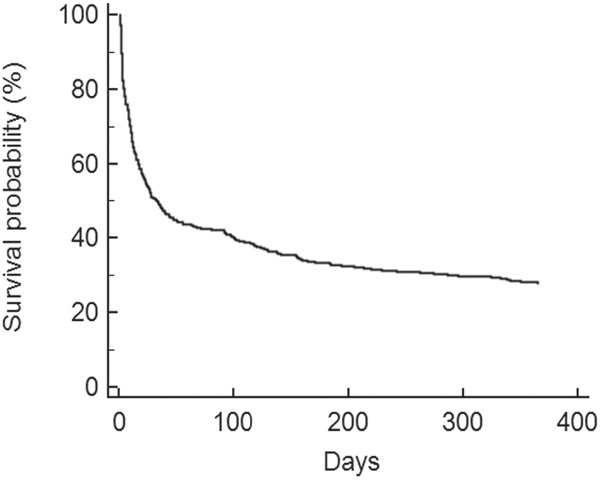
Overall Kaplan-Meier survival curve

Two hundred and seventy-one hematological malignancies (65%) were high-grade and 147 (35%) were low-grade. Table 3 summarizes a comparison of the patients according to their hematological malignancy. ICU and day-28 mortality for the whole cohort was 46% (194/418) and 49% (203/418), respectively. Mortality increased from 58% (242/418) at day-90 to 72% (302/418) at 1 year (Figure 1). The type of hematological malignancy did not influence either the short- or the long-term outcome (Table 3). By multivariate analysis, variables associated with day-28 mortality were IMV (OR, 7.17; 95% CI, 4.38-11.72), RRT (OR, 2.82; 95% CI, 1.60-4.99) and performance status (OR per point, 1.48; 95% CI, 1.16-1.89) (Table 1). Independent predictors of 1-year mortality were performance status, recurrence or progression status, neutropenia, IMV, and RRT (Table 4).

**Table 3:** Most common hematological malignancies-univariate comparison

	Acute Leukemia (n=239)	Myeloma (n=69)	Lymphoma (n=53)	p Value
Age^a^	52 ± 15	60 ± 13^d^	55 ± 14^e^	<0.001
Sex (male)^b^	136 (57)	43 (62)	32 (59)	0.69
Charlson score^a^	3.7 ± 1.7^e^	5.0 ± 2.4^d^	4.4 ± 2.2^d^^e^	<0.001
PS^a^	1.8 ± 1.0	2.1 ± 0.9^d^	1.9 ± 1.0	0.02
Newly diagnosed^b^	103 (43)	12 (17)^d^	11 (21)^d^	<0.0001
Autologous HSCT^b^	7 (3)	24 (35)^d^	11 (21)^d^	<0.0001
Allogeneic HSCT^b^	61 (26)	21 (30)	15 (28)	0.70
GVHD^b^	31 (13)	6 (9)	6 (11)	0.62
Neutropenia^b^	84 (35)	14 (20)^d^	10 (19)^d^	0.01
Respiratory distress^b^	122 (51)	28 (41)	21 (40)	0.15
SOFA score^a^	8 ± 3	8 ± 4	8 ± 4	0.13
IMV^b^^c^	108 (45)	31 (45)	25 (47)	0.96
RRT^b^^c^	46 (22)	20 (29)	11 (21)	0.22
Vasopressors^b^^c^	116 (49)	29 (42)	35 (66)^d^^e^	0.01
SAPS II^a^	55 ± 22	62 ± 28	57 ± 26	0.12
Day-28 mortality^b^	115 (48)	35 (51)	21 (40)	0.44
Day-90 mortality^b^	130 (54)	42 (61)	28 (53)	0.63
1-year mortality^b^	167 (70)	50 (72)	35 (67)	0.75

a Data expressed as mean ± SD.

b Data expressed as number (percentage).

c Within the first 48 hours.

d p<0.05 compared with leukemia group.

e p<0.05 compared with myeloma group. GVHD: graft versus host disease; HSCT: hematopoietic stem cell transplantation; IMV: invasive mechanical ventilation; PS: performance status; RRT: renal replacement therapy.

**Table 4:** Logistic regression analysis according to 1-year outcome

	Odds Ratio	95% CI	p Value
Age	1.01	0.99-1.03	0.42
Charlson score	1.12	0.93-1.36	0.22
PS (per point)	1.54	1.17-2.03	0.002
Recurrence/Progression	3.2	1.76-5.85	<0.001
Allogeneic HSCT	1.78	0.93-3.40	0.08
Neutropenia	1.82	1.02-3.27	0.04
IMV^a^	5.49	3.09-9.78	<0.0001
RRT^a^	3.24	1.44-7.25	0.004
Vasopressors^a^	1.00	0.58-1.70	0.99

a Organ support during the entire ICU stay. CI: confidence intervals; HSCT: hematopoietic stem cell transplantation; IMV: invasive mechanical ventilation; PS: performance status; RRT: renal replacement therapy.

End-of-life decisions were implemented in 78 patients (19%): 43 (10%) were intubated, 21 (5%) had neutropenia, and 21 (5%) were allogeneic HSCT recipients. These decisions included no escalation of treatment (not to start treatment if it becomes necessary), withholding (not to start necessary treatment) and withdrawal (to stop necessary treatment). Day-28 mortality for patients with treatment limitation decisions was 73% (57/78) versus 43% (146/340) for the population without “do not resuscitate” orders (p<0.001).

### Allogeneic hematopoietic stem-cell transplantation recipients

**Figure 2:**
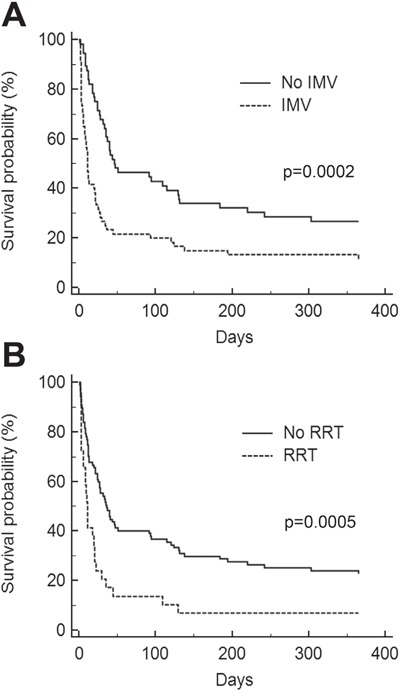
Kaplan-Meier survival curves for allogeneic hematopoietic stem-cell transplantation recipients **A.** Invasive mechanical ventilation (IMV). **B.** Renal replacement therapy (RRT).

One hundred and sixteen critically ill allogeneic HSCT recipients (28% of the whole cohort) were included in the study (Table 5). The source of stem cell was bone marrow, peripheral blood, and cord blood in 54% (n=63), 36% (n=42), and 10% of patients (n=12), respectively. Conditioning regimen was myeloablative in 107 patients (92%). Admission into the ICU occurred during the engraftment period for 40 patients (34%) and afterwards for 76 patients (66%). Sixty patients (52%) had GVHD. By univariate analysis, none of these transplantation-related characteristics significantly influenced the day-28 mortality. By multivariate analysis, only IMV and RRT were independently associated with short-term outcome (Table 6 and Figure 2).

**Table 5:** Patient characteristics according to their treatment/condition

	Allogeneic HSCT (n=116)	Neutropenia (n=124)	IMV (n=196)
Age^a^	46 ± 13	53 ± 15	55 ± 16
Sex(male)^b^	69 (59)	66 (53)	105 (54)
Charlson score^a^	3.2 ± 1.5	3.7 ± 1.7	4.1 ± 2.1
PS^a^	2.1 ± 1.0	1.8 ± 0.9	2.0 ± 1.0
Newly diagnosed^b^	NA	43 (35)	62 (32)
Autologous HSCT^b^	23 (20)	10 (8)	17 (9)
Allogeneic HSCT^b^	NA	38 (31)	60 (31)
GVHD^b^	60 (52)	8 (6)	32 (16)
Neutropenia^b^	38 (33)	NA	61 (31)
Respiratory distress^b^	61 (53)	54 (44)	105 (56)
SOFA^a^	8 ± 4	10 ± 3	10 ± 3
IMV^b^^c^	60 (52)	61 (49)	NA
RRT^b^^c^	29 (25)	30 (24)	69 (35)
Vasopressor^b^^c^	48 (41)	77 (62)	137 (70)
SAPS II^a^	53 ± 22	66 ± 23	70 ± 26
Day-28 mortality^b^	60 (52)	67 (54)	145 (74)
Day-90 mortality^b^	77 (66)	80 (65)	156 (80)
1-year mortality^b^	94 (81)	97 (78)	171 (87)

a Data expressed as mean ± SD.

b Data expressed as number (percentage).

c Within the first 48 hours. GVHD: graft versus host disease; HSCT: hematopoietic stem cell transplantation; IMV: invasive mechanical ventilation; PS: performance status; RRT: renal replacement therapy; SAPS II: simplified acute physiology score II; SOFA: sequential organ failure assessment.

**Table 6:** Day-28 logistic regression analysis according to the treatment/condition

	Allogeneic HSCT (n=116)	Neutropenia (n=124)	IMV (n=196)
Charlson score	NA	NA	NS
PS (per point)	NS	1.69 [1.04-2.76]^b^	NA
Neutropenia	NA	NA	NS
IMV^a^	4.09 [1.80-9.30]^c^	9.69 [3.86-24.33]^c^	NA
RRT^a^	3.78 [1.34-10.65]^b^	NS	2.08 [1.00-4.33]^b^
Vasopressors^a^	NA	NS	NA

Data expressed as mortality odds ratio [95% confidence intervals].

a Within the first 48 hours.

b p<0.05;

c p<0.001. IMV: invasive mechanical ventilation; NA: not applicable; NS: not significant; PS: performance status; RRT: renal replacement therapy.

### Neutropenic patients

**Figure 3:**
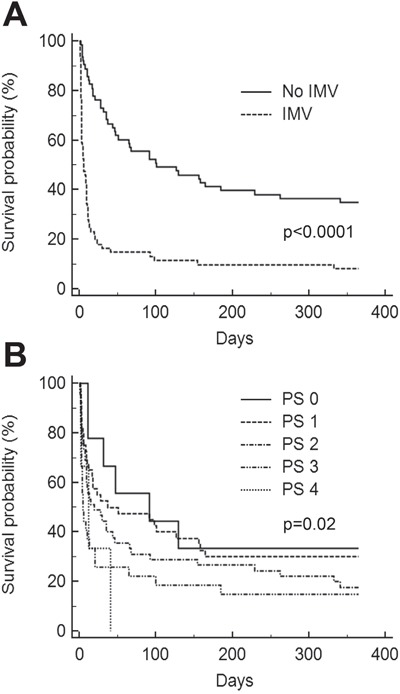
Kaplan-Meier survival curves for neutropenic patients **A.** Invasive mechanical ventilation (IMV). **B.** Performance status (PS).

Among the 124 patients with neutropenia (30% of the whole cohort) at ICU admission (see characteristics in Table 5), 85 (69%) had a white blood cell count lower than 0.1×10^9^ /L. Neutropenia followed a course of chemotherapy in 113 patients (91%). Thirty patients (24%) experienced neutropenia recovery at the end of their ICU stay. The characteristics of neutropenia had no influence on prognosis (data not shown). Independent predictors of day-28 mortality in this population were IMV and performance status (Table 6 and Figure 3).

### Invasive ventilated patients

**Figure 4:**
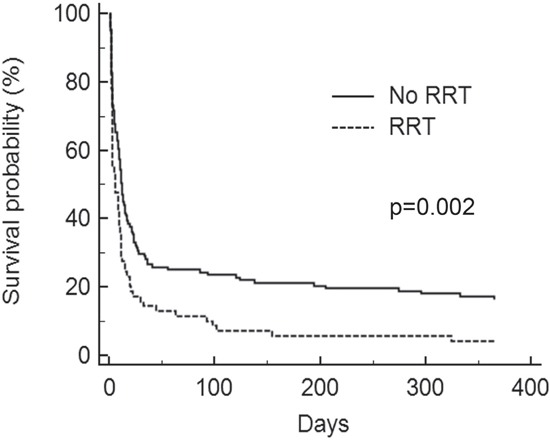
Kaplan-Meier survival curves for invasive ventilated patients RRT: renal replacement therapy.

During the study period, invasive ventilator support was provided in 196 patients (47% of the whole cohort, Table 5). It was the first line treatment in 131 patients (67%) while it followed NIMV failure in 65 patients (33%). One hundred and twenty five patients were initially treated with NIMV in the whole population, with a failure rate of 52% (65/125). Day-28 mortality was 33% (20/60) for NIMV success and 77% (50/65) in case of failure of the non invasive procedure. Second-line intubation did not result in a significant excess mortality. One hundred and seven patients (55%) fulfilled the ARDS criteria. The leading cause of intubation was pneumonia (n=98, 50%). Congestive heart failure was diagnosed in 10 patients (5%) while 19 patients (10%) were intubated for neurological reasons. Multivariate analysis showed that RRT was the only predictor of day-28 mortality in this population (Table 6 and Figure 4).

## DISCUSSION

Of the single-center studies currently available in the literature, this study is one of the largest involving patients with hematological malignancy admitted to an ICU. Additionally, these patients included a large proportion of allogeneic HSCT recipients. We report encouraging (even high) short- and long-term mortality rates that were stable over time. Invasive mechanical ventilation was the strongest predictor of day-28 mortality, whereas allogeneic HSCT or neutropenia did not influence the short-term prognosis of onco-hematological patients. Thus, we advocate that HSCT and neutropenia may no longer be considered as poor prognosis factors in this population of patients.

Our results show that approximately one in two critically ill patients with hematological malignancy died in the acute phase of medical care. This mortality rate is consistent with other recent studies, which found ICU mortality ranged between 34% and 61% [9–11, 19]. These results are even more promising as the severity scores of our patients (i.e. SOFA score and SAPS II) were higher than those previously reported [8, 12]. We found no differences among admission parameters, the type of malignancy, or ventilatory support strategies over the study period. This allowed us to study the cohort as a whole despite the 10-year period. Our ICU cares for more than thirty patients per year with hematological malignancy, which may lead to a greater understanding of specific complications and closer collaborations with hematologists. Indeed, Lecuyer et al. revealed a relationship between case volume and ICU outcome in hematological patients [20]. However, the 1-year mortality rate we observed remained higher than that recently reported by Azoulay et al. in a multicenter study [12]. In order to explain this difference, we could argue that SOFA score at admission in our cohort was higher and that our refusal rate of patients considered for ICU admission was very low. We acknowledge that one limit is that we did not have data on the ICU admission policy in hematologic ward, which could also impact the outcome in our cohort. Even if determinants of long-term outcome are often difficult to analyze, we can make an assumption. According to our liberal triage protocol, our admission criteria were based on the severity of the illness rather than on the stage of hematological malignancy. Corroborating data suggest that prognosis of malignancy does not predict ICU outcome [9, 21]. In the present work, one third of the patients admitted to the ICU had an uncontrolled malignancy, which was likely to affect the long-term outcome. Indeed, we showed that the stage of cancer was a strong predictor of 1-year mortality of onco-hematological patients. Thus, the subgroup of patients whose malignancy had relapsed or progressed may explain the gap between short- and long-term mortality. However, due to the lack of some information regarding the underlying malignancy, we could not exclude that other characteristics (e.g. cytogenetic data) may also have influenced 1-year outcome.

Our study confirms that invasive ventilation is the main factor that determines short-term outcome in onco-hematological patients. The need for intubation has been consistently found to be a factor of poor prognosis in this population [7–13]. Although the notion was controversial, we chose to analyse IMV as a surrogate of respiratory failure rather than a modifiable risk factor. Therefore, we focused on this subgroup with a high risk of mortality and investigated a large cohort of invasively ventilated patients. As previously described, acute respiratory failure was the main reason of admission to the ICU for these patients [8, 9, 11, 12] The ventilation support strategy appears decisive, with mortality rates dramatically different according to the need for intubation. We have described a day-28 mortality of 74%, which is consistent with previous reports. Molina et al. recently showed a 75% mortality rate in 248 invasively ventilated patients with hematological malignancy [22]. In a large cohort of 717 cancer patients, Azevedo et al. also found that 73% of patients requiring intubation died in the ICU [23]. High scores for the severity of the illness, the presence of ARDS criteria in most patients, and a low rate of congestive heart failure etiology may explain the poor survival rate we observed. Although NIMV has been shown to decrease mortality, patients with hematological malignancies experience a high failure rate for this technique [5, 7, 22, 23]. In our study, as in the literature, nearly half of patients initially treated with NIMV required intubation. Few studies have specifically described prognostic factors for patients with hematological malignancy requiring invasive ventilation [22, 24]. Recently, Molina et al. found that allogeneic HSCT, NIMV failure, and APACHE II were independent risks factors that increased mortality [22]. In our study, only RRT was associated with mortality and the presence of allogeneic HSCT did not affect the outcome of ventilated patients.

Among patients with hematological malignancy, allogeneic HSCT recipients are considered to be those who would benefit the least from intensive care treatment. They are usually younger and require higher ICU resources than the other patients [25, 26]. Their prognosis may be worsened by specific complications, such as GVHD, which require increased immunosuppressive therapy [27]. Over the past twenty years, admissions to the ICU and the initiation of organ support for these patients have raised ethical concerns. In 1996, Rubenfield et al. reported a 100% mortality rate among mechanically ventilated recipients of bone marrow transplants, questioning the merits of such a technique for these patients [28]. Our results have shown that, today, better survival rates can be achieved for onco-hematological patients with or without allogeneic HSCT, even if they are ventilated. We can assume that early treatment of the respiratory distress, which avoids the delay of intubation, and an adequate triaging strategy can lead to this result. The most recent studies have reported hospital mortality rates ranging from 52% to 68% for allogeneic HSCT recipients admitted to ICUs [27, 28]. Here, we reported a day-28 mortality rate of 52%, which appears to be an encouraging result since half of the patients were invasively ventilated. In 2006, Pene et al. found mechanical ventilation, corticosteroid treatment for GVHD, and bilirubin levels to be independently associated with mortality [27]. In our cohort of HSCT recipients, only IMV and RRT were predictive of mortality. Nevertheless, we have to underline that a lack of statistical power could be a limit for the analysis of small subgroups, such as patients with GVHD. Thus, we confirm, in the population of allogeneic HSCT recipients, that prognostic factors are mostly related to organ support (i.e. surrogates of organ failure), whereas transplantation characteristics do not appear to impact short-term outcome.

Various studies have also associated neutropenia with mortality among onco-hematological patients admitted to ICUs [9, 30, 31]. As a result, clinicians are sometimes reluctant to admit neutropenic patients into the ICU and/or to initiate organ support. In fact, literature about critically ill cancer patients gives conflicting results about the impact of neutropenia [9, 12, 30, 31]. Indeed, Souza-Dantas et al., in a match-case controlled study, provided evidence that the presence of neutropenia was no longer associated with worse outcome among cancer patients [32]. In a large prospective cohort of onco-hematological patients, Azoulay et al. confirmed that neutropenia was not associated with short-term outcome [12]. Our results, consistent with these recent studies, contribute to the closing of the debate [12, 32]. Low levels of white blood cells should be added to the list of classic mortality predictors which are no longer relevant, such as the type of malignancy, the disease status, recent bacteriemia, or chemotherapy [9, 33]. We reported here a 54% mortality rate at day-28 for this subgroup of patients. Legrand et al., in a cohort of 428 critically ill patients with neutropenia, reported a 50% hospital mortality rate, decreasing along the study period [34]. This is consistent with the mortality rates we observed, even if we did not observe an improvement in survival over the ten-year period we studied. In addition to IMV, performance status was also an independent predictor of day-28 mortality for our cohort. Indeed functional assessment on admission was found to be a key prognostic factor in some previous studies [12]. Interestingly, performance status is all the more relevant as it is easily assessed for all patients upon admission in all patients.

In summary, our results are both encouraging and disturbing. Our study confirms a low survival rate for patients with hematological malignancy requiring IMV. Our data also support the fact that neutropenia is no longer associated with short-term mortality and that an encouraging mortality rate can be achieved for HSCT recipients. Ventilatory support strategies definitely play a key role in patient outcome and efforts should be made to limit the need for intubation without delaying it.

## MATERIALS AND METHODS

### Design, setting and patients

The ethics committee, *Comité de Protection des Personnes Sud-Est II*, approved this retrospective non-interventional study. The need for consent was waived given the retrospective design of the project. The study was performed in compliance with the ethical standards of the Declaration of Helsinki and according to French laws.

The study was conducted in a 15-bed university-affiliated adult medical ICU. From January 2002 to December 2011, all adult patients (≥18 years old) admitted to the ICU with hematological malignancy were included. In our hospital, all patients with cancer were eligible for ICU admission unless explicit advanced directives were present. Admission policy was not restrictive.

### Data collection and definitions

Variables collected within the first 24 h of ICU admission included: age, gender, comorbidities according to the Charlson score [14], performance status [15], type of malignancy, disease status, bone marrow transplant status (type, conditioning regimen), presence of graft versus host disease (GVHD), neutropenia, reason for admission, and severity of illness (according to both SOFA score [16] and SAPS II [17]). Type of organ support (IMV, NIMV, renal replacement therapy, RRT and vasopressors) was collected within the first 48 hours and during the entire ICU stay. Mortality was evaluated at day-28, day-90, and 1 year.

As previously described, high-grade malignancies included acute leukemia and aggressive lymphoma, whereas low-grade malignancies included chronic leukemia, indolent lymphoma, myeloma, Waldenström macroglobulinemia, and aplastic anemia [9]. Disease status was defined by a hematologist prior to ICU admission and included newly diagnosed (<4 weeks), controlled/remission, or recurrence/progression. Neutropenia was defined as a leukocyte cell count <0.5.10^9^/L. Bone marrow transplants were autologous or allogeneic. The recorded reason for admission was based on the main symptom upon being admitted into the ICU. According to the SOFA, each organ failure was defined by a score >2 [16]. Diagnosis of ARDS was made in accordance with the 1994 American-European consensus-conference [18].

### Statistical analysis

Values are expressed as mean ± standard deviation (SD) and number (percentage), as appropriate.

Clinical data were first compared in two-year periods over the timeframe of the study. Data were also compared according to the type of hematological malignancy. Comparisons were carried out using a one-way analysis of variance (ANOVA) for continuous data and the chi-squared test for categorical variables.

Prognostic variables in determining day-28 and 1-year mortality were assessed by univariate analyses using an unpaired Student's t-test for continuous data and the chi-squared test or fisher's exact test for categorical data, as appropriate. All variables with p<0.15 were included in multivariate analysis using a logistic regression model. As we chose to evaluate the impact of organ support, the reason for admission, SOFA, and SAPS 2 were excluded from the model because of redundancy. Odds ratios (OR) were estimated with 95% confidence intervals (95% CI). Initial analyses included the whole cohort. Depending on day-28 outcome, patients were also analyzed according to treatment/condition details (allogeneic HSCT, neutropenia, IMV). Each of these three subgroups underwent univariate and multivariate analysis similar to that described above. These analyses were performed independently but the groups were not mutually exclusive.

Time to death for the whole cohort and for the 3 predefined subgroups of patients were modeled by means of Kaplan-Meier estimates, and differences were compared by use of the log-rank test.

Statistical calculations were performed using Medcalc Software version 7.4.3.0 (Mariarke, Belgium). Differences were considered significant when p<0.05.
